# Case Report: Acute onset hemiparesis in a young man: do not miss Crohn’s disease

**DOI:** 10.3389/fimmu.2025.1662213

**Published:** 2025-09-02

**Authors:** Virginia Iacobelli, Simone Tagliabue, Beatrice Modello, Daniele Velardo, Elena Abati, Fabio Triulzi, Giacomo Pietro Comi, Stefania Corti, Delia Gagliardi, Mosè Parisi

**Affiliations:** ^1^ Department of Pathophysiology and Transplantation (DEPT), University of Milan, Milan, Italy; ^2^ Neuroradiology Unit, Fondazione IRCCS Ca’ Granda Ospedale Maggiore Policlinico, Milan, Italy; ^3^ Neurology Unit, Fondazione IRCCS Ca’ Granda Ospedale Maggiore Policlinico, Dino Ferrari Centre, Department of Neuroscience and Mental Health, Milan, Italy; ^4^ Neuromuscular and Rare Diseases Unit, Fondazione IRCCS Ca’ Granda Ospedale Maggiore Policlinico, Dino Ferrari Centre, Department of Neuroscience and Mental Health, Milan, Italy

**Keywords:** Crohn’s disease, inflammatory bowel disease, cerebral vasculitis, juvenile stroke, intracranial vessel wall imaging

## Abstract

Crohn’s disease (CD) is a chronic inflammatory bowel disease that may include neurological complications, besides gastrointestinal manifestations. Although cerebrovascular complications are commonly reported, cerebral vasculitis remains an exceedingly rare occurrence and only a limited number of cases have been described. We present the case of a 35-year-old man with CD who presented with acute onset of right-sided hemiparesis, hemiataxia and paresthesias. Laboratory data showed an inflammatory profile. Contrast-enhanced brain magnetic resonance angiography (MRA) with vessel wall imaging well demonstrated focal areas of contrast enhancement in the perforating arteries and distal arterial branches of intracranial vessels, raising the suspicion of a vasculitic process. The patient was then started on high-dose steroid therapy with immediate improvement of the neurological condition. Follow-up brain MRA revealed a significant reduction of the focal contrast-enhancing alterations. If not accurately identified and promptly treated, vasculitic processes may lead to significant disabilities in young patients and should be considered in the differential etiologies of juvenile stroke since symptoms can improve with immunosuppressive treatment. This case highlights the broad spectrum of possible etiologies to be considered in a young patient presenting with an acute onset neurological syndrome and provides a stepwise approach to developing a comprehensive differential diagnosis.

## Introduction

Inflammatory bowel diseases (IBD) are a group of chronic inflammatory disorders primarily affecting the gastrointestinal tract. IBD comprise two major forms, ulcerative colitis and Crohn’s disease (CD). CD is characterized by a relapsing-remitting course and may affect any part of the digestive system. In addition to the classical gastrointestinal symptoms, which include abdominal pain, diarrhea, and weight loss, patients with IBD frequently experience a range of extraintestinal manifestations occurring in the joints, mouth, eyes, skin and liver, besides some more uncommon manifestations. For instance, some extraintestinal manifestations in IBD include peripheral arthritis, axial arthropathies, erythema nodosum, pyoderma gangrenosum, uveitis, episcleritis, oral aphthous ulcers, hepatobiliary disorders such as primary sclerosing cholangitis, and less frequently, neurological complications (1, 2).

Neurological involvement in IBD is a rare condition that may affect both the central and the peripheral nervous system. Peripheral neuropathy, including both sensory and motor disturbances, can occur in the context of CD, whereas myopathic disorders have also been reported (3, 4). Central nervous system involvement is less common, and cerebrovascular complications are the most frequently reported, particularly in ulcerative colitis (5–7). Nevertheless, cerebral vasculitis secondary to CD remains an exceedingly rare occurrence and only a limited number of cases have been described (8–11). Within this limited literature, reported cases have predominantly involved young to middle-aged adults, typically between the third and fifth decades of life, although rare occurrences in pediatric or elderly individuals have also been documented (11–14). Gender distribution could suggest a slight female predominance, but this observation remains anecdotal and lacks robust epidemiological confirmation.

In this scenario, prompt recognition and treatment are crucial in preventing major morbidity and minimizing long-term disability, particularly in young patients.

We present a case of cerebral vasculitis in a young patient with CD providing a stepwise approach to develop the appropriate and accurate diagnosis. The rapid resolution of the neurological condition after the initiation of immunosuppressive treatment underscores the importance of a correct therapeutic strategy for achieving a favorable outcome in these patients.

## Case presentation

A 35-year-old man presented to the emergency department one hour after the onset of acute weakness and paresthesias on the right side of his body. He reported lifting weights a few hours before the onset of symptoms and a single episode of diarrhea two days before, without fever or other associated systemic symptoms.

Past medical history included a diagnosis of Crohn’s disease at the age of 11; he was initially treated with Azathioprine 1 mg/kg a day for the first 7 years, followed by subcutaneous injections of Adalimumab (anti-TNFα human recombinant monoclonal antibody), which was continued until clinical remission was achieved. Treatment was discontinued four years prior to the onset of neurological symptoms.

The patient was a mild smoker (5 cigarettes/day) for the last 15 years. No additional vascular risk factors nor family history of cardiovascular conditions or inflammatory/autoimmune diseases emerged.

At the emergency department, the patient exhibited blood pressure values of 150/95 mmHg, preserved respiratory mechanics, and was afebrile. Electrocardiography revealed sinus tachycardia with a heart rate of 110 bpm. Urgent blood tests, including complete blood count with leukocyte differential, blood glucose, creatinine, C-reactive protein (CRP), sodium, potassium, aspartate aminotransferase (AST), alanine aminotransferase (ALT), prothrombin time (PT), and activated partial thromboplastin time (aPTT), were within normal ranges except for a mildly elevated CRP of 1.20 mg/dL (normal values <0.5 mg/dL). On initial neurological examination, the patient showed right facial palsy sparing the forehead, mild weakness of the right limbs, predominantly involving upper limb extensors and lower limb flexors and graded 4/5 on the Medical Research Council (MRC) scale, and right-sided tingling paresthesias. Cerebellar testing revealed slight incoordination and dysmetria on finger-to-nose and heel-to-shin maneuvers. Deep tendon reflexes were present and symmetric in all four limbs.

Acute onset of central symptoms suggests an abrupt process involving the left cerebral hemisphere. As a primary hypothesis, it is imperative to evaluate whether the neurological symptoms are due to ischemic or hemorrhagic stroke, whereas a seizure is unlikely due to the absence of positive symptoms and previous history of epilepsy. Despite demyelinating lesions and brain tumors typically display insidious presentations, they should be considered as possible alternative diagnoses as more rapid disease onsets can also occur.

The patient needed urgent brain imaging to guide immediate management. A brain CT scan showed a small focal hyperdensity in the left sublenticular region surrounded by perilesional edema. No vascular stenosis, occlusions, aneurysms, dissections, or malformations were revealed by the CT angiogram. Contrast-enhanced imaging of the brain parenchyma demonstrated enhancement of the sublenticular hyperdensity and left thalamo-mesencephalic region.

**Figure 1:**
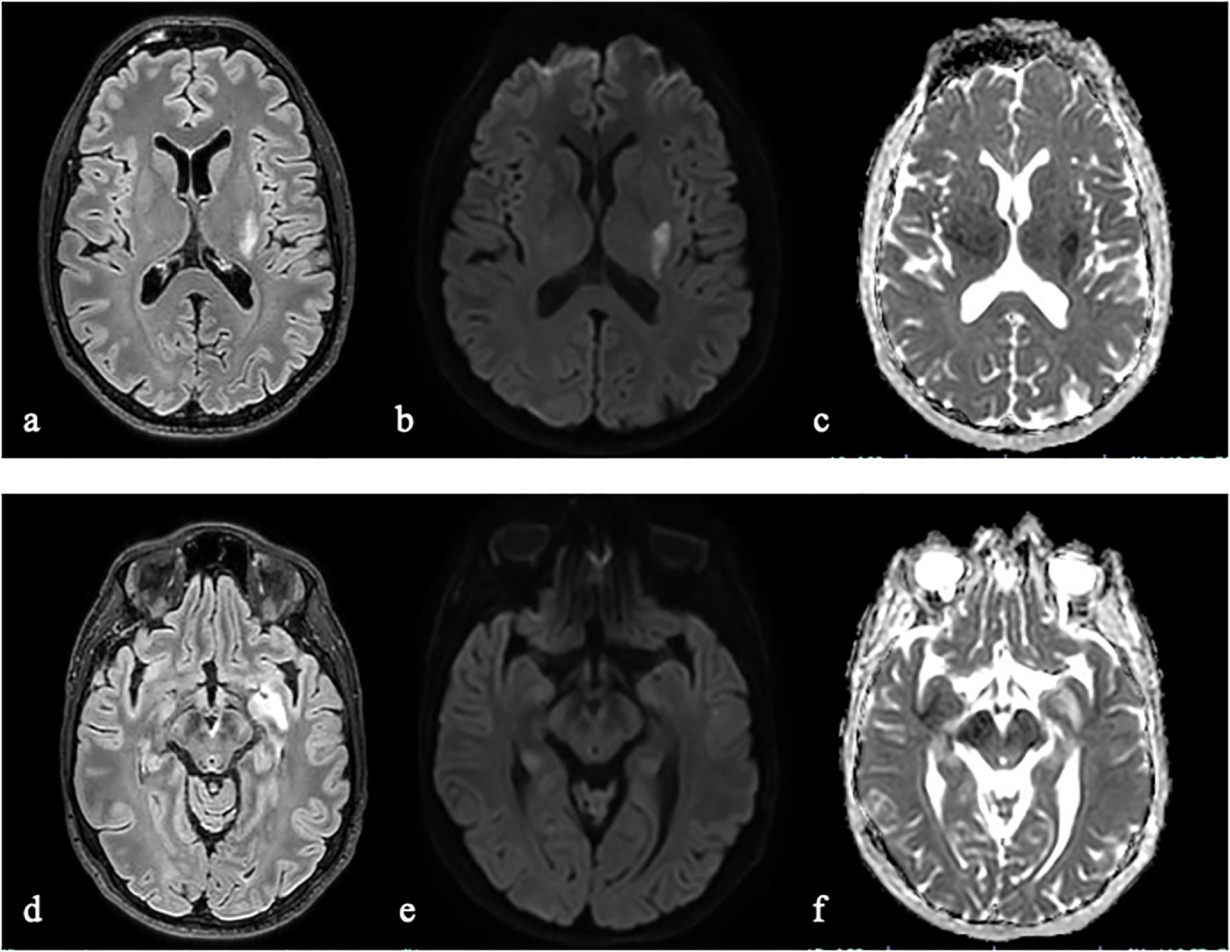
Brain MRI demonstrated an acute left lenticular hyperintense lesion on fluid-attenuated inversion recovery (FLAIR) imaging **(a)** with a diffusion restriction pattern, characterized by hyperintensity on diffusion-weighted imaging (DWI, **b**) and corresponding hypointensity on apparent diffusion coefficient (ADC) maps **(c)**. Additionally, MRI identified a hyperintense lesion on FLAIR within the left sublenticular-temporal region **(d)**, exhibiting mild hyperintensity on DWI **(e)** and hyperintensity on ADC maps **(f)**, suggestive of vasogenic edema.

Contrast-enhanced brain MRI (**Figure 1**) confirmed the alteration in the left sublenticular region characterized by focal hyperintense signal in T1, T2/FLAIR, and SWI sequences, and peripheral ring enhancement without diffusion restriction. Additionally, a T2/FLAIR hyperintensity with diffusion restriction in the dorsal portions of the left putamen and globus pallidus, as well as T2/FLAIR hyperintensity without diffusion restriction in the left cerebral peduncle, were observed. The MRI findings were initially interpreted as ischemic lesions possibly related to septic microembolism or, less likely, as inflammatory or neoplastic in nature.

Revisiting clinical history, no clues pointing towards infective endocarditis, or a neoplastic process were recognized. The patient did not experience fever, fatigue or arthralgias in the days prior to admission. No recent dental procedures or known immunosuppressive factors were present. No unexplained weight loss over the past year was reported.

To rule out the infectious hypothesis, blood cultures were collected, along with antibody titers for *Epstein-Barr virus* (EBV), *Cytomegalovirus* (CMV), *Toxoplasma gondii*, *Treponema pallidum*, *hepatitis C virus* (HCV) and *human immunodeficiency virus* (HIV), surface antigen of the *hepatitis B virus* (HBV), *Cryptococcus neoformans* capsular antigen, interferon gamma release assay (IGRA) for *Mycobacterium tuberculosis*. Instrumental investigations to search for embolic sources included transesophageal echocardiogram at rest and after Valsalva maneuver, dental panoramic radiograph, and CT scan of chest and abdomen, all yielding negative results. As concerns the neoplastic suspicion, a total body positron emission tomography (PET) with fluorodeoxyglucose (FDG) did not display any lesion with elevated glucose metabolism, while a reduced radiotracer uptake was found in the brain areas corresponding to the MRI hyperintense signals.

Finally, a lumbar puncture was performed. The cerebrospinal fluid (CSF) analysis revealed 42 cells/uL. Cytological assessment showed numerous activated lymphocytes along with some monocytes and neutrophilic granulocytes. Glucose and protein levels were within normal limits. CSF culture and polymerase chain reaction (PCR) test for neurotropic pathogens (*Herpes simplex virus* (HSV) *type 1* and *type 2* DNA, *Varicella zoster virus* (VZV) DNA, *Human herpesvirus 6* (HHV-6) DNA, *Enterovirus* RNA, *Parechovirus* RNA, *Neisseria meningitidis* DNA, *Haemophilus influenzae* DNA, *Streptococcus pneumoniae* DNA, group B *Strep.* DNA, *E. coli K1* DNA, *Listeria Monocytogenes* DNA, *Cryptococcus neoformans* DNA, *Streptococcus pyogenes* DNA, *Mycoplasma pneumoniae* DNA) were negative. Oligoclonal bands were absent in the CSF.

**Figure 2:**
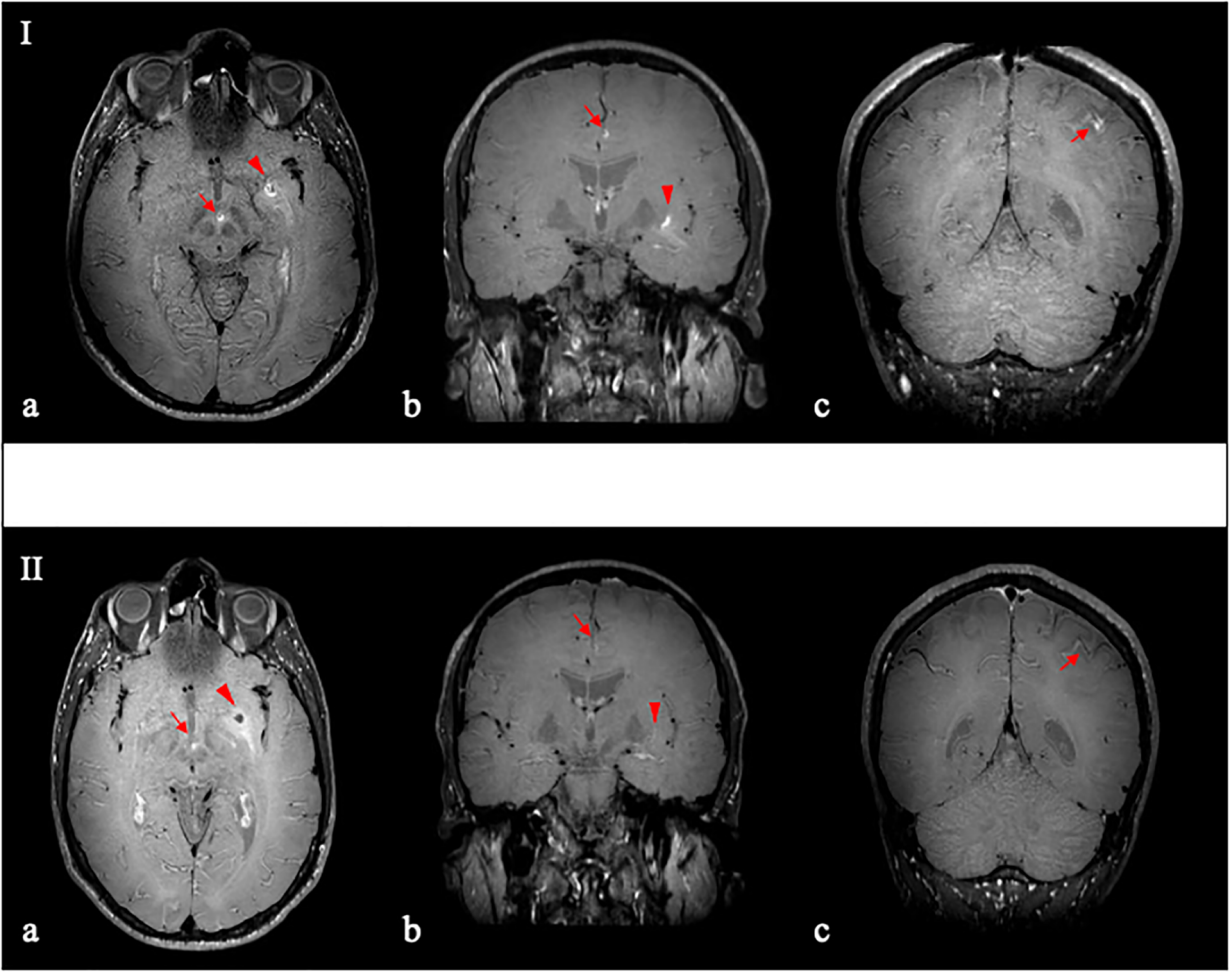
Contrast-enhanced vessel wall imaging (VW–MRI), before (I) and after (II) immunosuppressive therapy. Pre-treatment VW–MRI (I) demonstrated vessel wall thickening and enhancement in the interpeduncular perforating arteries (I–**a**; arrow) and the left middle cerebral artery (I–**a,b**; arrowhead); and in the distal branches of the left anterior cerebral artery (I–**b**; arrow) and the ipsilateral middle cerebral artery (I–**c**; red arrow). After 3 months of immunosuppressive therapy VW–MRI (II–**a**–**c**) revealed a marked reduction in contrast enhancement of the aforementioned arterial walls.

Given these results, we considered inflammatory conditions, such as vasculitic processes, among the etiologies of juvenile stroke. In this view, we performed a contrast-enhanced brain MR angiography (MRA) with vessel wall imaging, revealing focal areas of contrast enhancement in the wall of perforating arteries of the midbrain and the left middle cerebral artery, in the wall of A3-A4 segment of the left anterior cerebral artery and in the post-rolandic sulcal branch on the same side (**Figure 2I-a-c**). The areas of enhancement adjacent to perforating arteries and distal arterial branches, in the context of ischemic and/or edematous alterations, raised the suspicion of inflammatory/vasculitic changes.

Genetic testing for Factor V Leiden (G1691A) and the prothrombin gene mutation (G20210A) yielded negative results. Autoimmune screening panel, which included rheumatoid factor, anti-gliadin antibodies (AGA), antineutrophil cytoplasmic antibodies (ANCA), antinuclear antibodies (ANA), extractable nuclear antigens (ENA), antiphospholipid antibodies, and anti-double stranded DNA antibodies (anti-dsDNA), showed positive ANA at a high titer (1/640) with homogeneous pattern. Blood tests confirmed a mild increase in CRP to 0.6 mg/dL and revealed a slightly elevated complement protein C4 of 61 mg/dL (reference range: 10–40 mg/dL), while C3 and the erythrocyte sedimentation rate (ESR) resulted within normal limits.

Finally, the potential contribution of prior treatment with Adalimumab was taken into consideration. Anti-TNFα agents are acknowledged as possible triggers of drug-induced vasculitis; however, evidence from the literature indicates that such events generally arise during active treatment or shortly thereafter (15, 16). In the present case, therapy had been discontinued four years before symptom onset, making a direct causal association highly improbable.

Based on the exclusion of alternative stroke etiologies, the integration of MRI/MRA findings, and the inflammatory profile on the CSF, we suspected cerebral vasculitis. Since several cases have been previously reported (8, 9, 11), we suspected that the vasculitis was secondary to Crohn’s disease. A brain biopsy was not performed due to the deep location of the lesions and the supportive evidence provided by advanced neuroimaging, which demonstrated vessel wall involvement consistent with vasculitis.

The patient was thus initiated on high-dose steroid therapy, starting with intravenous methylprednisolone 1g per day for three days, followed by oral prednisone 50 mg per day in combination with maintenance immunosuppressive therapy with azathioprine 100 mg per day. The neurological condition improved rapidly, leading to the complete disappearance of weakness, incoordination and paresthesias. In the following weeks, the steroid therapy was gradually tapered, while the azathioprine dosage was maintained at 150 mg per day, with persistence of clinical benefit.

After 3 months of immunosuppressive therapy, we repeated a brain MRA with contrast-enhanced vessel wall imaging, demonstrating a significant reduction of the focal contrast-enhancing alterations in the wall of the aforementioned intracranial vessels (**Figure 2II-a-c**).

## Discussion

Crohn’s disease (CD), along with ulcerative colitis, is a chronic inflammatory bowel disease (IBD) characterized by a relapsing-remitting course. CD is an autoimmune condition that can affect any part of the digestive system. Besides gastrointestinal manifestations, which in CD may include abdominal pain, fever, bowel habit changes, or perianal disease, extraintestinal manifestations involving the joints, mouth, eyes, skin, liver, and the central and peripheral nervous system have also been described.

The reported incidence of neurological manifestations in IBD varies significantly, ranging from 0.25% to 47.5% (17–20). Importantly, neurologic involvement can precede gastrointestinal symptoms of IBD, and it is not necessarily linked to intestinal inflammatory activity (21). In fact, vasculitic manifestations and other extraintestinal complications of IBD may occur during periods of remission, underscoring the need for vigilance even in clinically inactive disease (22). The pathophysiology is mostly immune-mediated, but contributory mechanisms can be represented by nutritional and metabolic disorders, prothrombotic state and side-effects of medications (23).

Central nervous system complications in IBD encompass a wide spectrum, including cerebrovascular and demyelinating disorders, progressive myelopathy, central nervous system infections, epilepsy and encephalopathy. Although cerebrovascular complications are the most commonly reported, cerebral vasculitis remains an exceedingly rare occurrence and only a limited number of cases, small case series, and reviews have been published (8, 9, 11, 22, 24). The clinical manifestations of cerebral vasculitis can have a sudden or a subacute onset and include headache (25), cranial nerve palsies, focal deficits, seizures and encephalopathy (22, 24–26). Brain MRI typically reveals non-specific multifocal and bilateral gray and white matter lesions, but special techniques such as Black Blood MRI, contrast-enhanced vessel MRI, or catheter angiography may be helpful in order to visualize the inflammation of the vessel wall directly (9, 27). Notably, the presence of elevated inflammatory markers in CSF can support the diagnosis (27). Finally, brain biopsy is still the gold standard for diagnosis of cerebral vasculitis, although it is an invasive procedure (28).

This case report illustrates a paradigmatic instance in which a patient with CD – clinically quiescent and off immunosuppressive or immunomodulatory therapy – developed a cerebrovascular event in the absence of concurrent infectious processes or systemic inflammatory activity. Notably, the possibility of an alternative or coexisting etiology has been carefully acknowledged and systematically ruled out through a combination of neuroimaging, CSF analysis, serologic tests, and whole-body FDG-PET. Although the coexistence of primary angiitis of the central nervous system (PACNS) and CD cannot be completely excluded, the presence of an established systemic autoimmune disease, current diagnostic criteria and supportive multimodal evidence makes secondary vasculitis the most plausible interpretation. The pattern of cerebral involvement, the presence of inflammatory markers in CSF, the focal enhancement of intracranial vessel walls supported by high-resolution specialized MRI technique, and the prompt and sustained response to immunosuppressive treatment collectively support the diagnosis of an inflammatory vasculopathy involving small- to medium-sized intracranial arteries.

When comparing our findings with previously reported cases, most descriptions in the literature involve large territorial cerebral infarctions resulting from vasculitis affecting large- to medium-sized vessels. In contrast, this case report highlights the uncommon occurrence of deep, well-circumscribed lesions attributable to selective involvement of small cerebral vessels. Furthermore, the present case is noteworthy for its onset during prolonged clinical remission of CD. This temporal dissociation challenges the conventional assumption that cerebral vasculitis in CD predominantly coincides with gastrointestinal exacerbations and underscores the importance of maintaining a high index of suspicion even in the context of apparently quiescent disease. From a therapeutic standpoint, our case corroborates previous evidence indicating that high-dose corticosteroids remain the mainstay of treatment, frequently resulting in rapid neurological improvement and radiological regression.

Although histopathological confirmation was not obtained, the integration of clinical, laboratory, and radiologic findings makes a compelling case for an immune-mediated vascular process in the context of a systemic autoimmune disorder, as CD. Future investigations should prioritize the validation of multimodal diagnostic approaches and their integration into standardized clinical pathways, with the ultimate goal of reducing reliance on invasive procedures, such as brain biopsy.

This case contributes to the growing recognition of cerebral vasculitis as a potential, though rare, neurological manifestation of CD, and underscores the importance of early consideration and intervention to improve clinical outcomes.

## Conclusion

This case highlights the broad spectrum of etiologies that must be considered when evaluating a young patient presenting with an acute onset hemiparesis. Although a rare cause of juvenile stroke, it is essential to consider cerebral vasculitis as a potential etiology and account its heterogeneous clinical presentations. Cerebral vasculitis in patients with CD represents a rare but critical neurological complication in the spectrum of IBD-associated manifestations. In this report, the MRI/MRA features and the inflammatory profile on the CSF, despite the absence of a clinically evident intestinal inflammatory activity, were crucial to provide the appropriate diagnosis and the consequently effective therapeutic strategy, making more invasive diagnostic techniques, such as brain biopsy, not necessary.

Importantly, this case highlights the imperative of recognizing neurological symptoms as potential extraintestinal manifestations of systemic autoimmune disorders such as CD, maintaining a high index of clinical suspicion even during remission phases of the underlying autoimmune condition. Although uncommon, cerebral vasculitis may present with acute focal neurological deficits, often mimicking more prevalent cerebrovascular pathologies. Prompt identification of this immune-mediated etiology is crucial for the initiation of targeted immunosuppressive therapy, with the goal of preventing irreversible neurological sequelae, particularly in younger individuals.

## Data Availability

The raw data supporting the conclusions of this article will be made available by the authors, without undue reservation.
